# Enhancing electrical conductivity in zirconium-doped SiC ceramics through synergistic effects of crystal structure and free carbon control†

**DOI:** 10.1039/d4ra06633b

**Published:** 2024-10-28

**Authors:** Jiaojiao Jiang, Siwei Ying, Chunxiao Wu, Chao Yang

**Affiliations:** a State Key Laboratory of Biochemical Engineering, Key Laboratory of Biopharmaceutical Preparation and Delivery, Key Laboratory of Green Process and Engineering, Institute of Process Engineering, Chinese Academy of Sciences Beijing 100190 China cxwu@ipe.ac.cn chaoyang@ipe.ac.cn; b School of Chemical Engineering, University of Chinese Academy of Sciences Beijing 100049 China; c Hubei Three Gorges Laboratory Yichang 443100 China; d State Key Laboratory of Physical Chemistry of Solid Surfaces, Collaborative Innovation Center of Chemistry for Energy Materials (iChEM), College of Chemistry and Chemical Engineering, Xiamen University Xiamen 361005 China

## Abstract

Polymer-derived ceramics (PDCs) have risen to prominence for applications in electrochemical energy storage, electromagnetic absorbing, and sensing materials, among others. However, a multitude of critical properties in PDCs are still limited by their intrinsic poor electrical conductivity. Herein, novel vinyl and zirconium-modified polycarbosilane precursors with improved electrical conductivity were synthesized through a Grignard coupling reaction of vinyl magnesium chloride and zirconocene dichloride, followed by the insertion polymerization with dichlorodimethylsilane and sodium. Our findings reveal a complex structural evolution from amorphous ZrO_2_ to distinct phases like *m*-ZrO_2_, *t*-ZrO_2_, and ZrC. This transformation significantly impacts the distribution and morphology of free carbon ribbons, influencing the material conductive network and resulting in an enhanced electrical conductivity of 0.28 S cm^−1^ and reduced bandgap. Through density functional theory analysis, a unique interaction between the energy bands of the carbon ribbons and the native cell is discovered, leading to a narrowed energy bandgap and conductive behavior. This advancement not only offers insight into the material structure–conductivity relationship but also opens new avenues for applications such as lithium battery anodes.

## Abstract 2

Synthesis and structural evolution of vinyl/Zr-modified SiC ceramics: enhanced conductivity, dree carbon dynamics, narrowed bandgap *via* nanoscale modification and DFT analysis, with potential applications in lithium-ion batteries and beyond.

## Graphical abstract

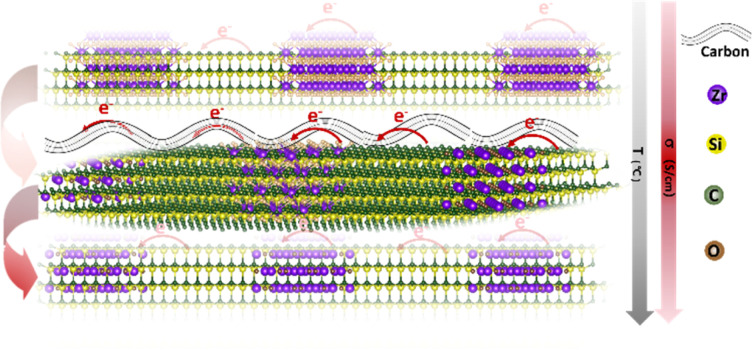

## 1. Introductions

Si-based advanced ceramics have consistently held a prominent position in various industries, such as electronics, aerospace, energy, and biomaterials, due to their exceptional mechanical, thermal, and electrical properties.^1–7^ The ability to tailor these materials for specific applications has sparked considerable interest, driven by the demand for enhanced performance and functionality. Among the various properties of these ceramics, electrical conductivity has emerged as a critical parameter for a wide range of applications, from electromagnetic interference shielding to advanced sensing technologies.^8–13^ This has led to a concerted effort to modify the microstructure and composition of these materials to meet the evolving requirements of these high-tech applications.^14–17^

Polymer-derived ceramics (PDCs) have gained prominence due to their unique properties and versatility in processing, making them suitable for applications beyond electrochemical energy storage,^16–21^ including electromagnetic absorbing^22–26^ and sensing.^27^ The electrical conductivity of PDCs, a key performance attribute, can be finely tuned through the molecular design of the precursors, highlighting the importance of precursor optimization in achieving desired nanoscale properties.^27–32^ However, it's worth noting that the inherently low electrical conductivity of PDCs limits their performance in high-tech applications, such as lithium-ion batteries.^33–37^ To enhance the electrochemical performance, numerous methods have been extensively studied, leading to significant progress.^38–43^ For example, Yang *et al.*^38^ developed a novel composite Li-ion battery cathode material, 1,4DHAQ&ZIF-8C, which improved electrode conductivity through its embedded microstructure and effective chemical adsorption, providing an integrated solution for light conversion and electricity storage.

In the case of PDCs, optimizing electrical conductivity involves complex multiphase and compositional transformations, a process that remains challenging and lacks comprehensive research. A critical aspect of this optimization is the *in situ* formation of “free carbon”, which plays a significant role in improving the conductivity of these materials. This carbon phase, derived from the hydrocarbon groups attached to the precursor backbone, plays a crucial role in enhancing electrical conductivity. The relationship between the precursor molecular structure and the resulting free carbon content and distribution is well-established, with studies indicating that unsaturated R groups in the precursor lead to higher free carbon yields.^44^ Although initial research, such as that by Su *et al.*,^45^ focused on the application of polymer-derived SiCN in Li-ion battery (LIB) anodes, the broader implications for electrical conductivity have become a focal point of recent studies. Kaspar *et al.*^46^ proved that a carbon content exceeding 20 wt% in SiOC materials significantly boosts electrical conductivity and cycling stability, underscoring the potential of carbon-rich materials for a wide array of applications.^47^ Luan *et al.*^48^ demonstrated that both the free carbon content and the degree of graphitization influence the conductivity of SiBCN coatings, which tends to decrease as the pyrolysis temperature increases. These studies consistently highlight that the presence of a free carbon phase significantly enhances electrochemical performance, and that the molecular structure of the precursor plays a crucial role in determining this performance. Notably, extensive research suggests that annealing temperatures are critical for structural evolution and nanoscale control of materials, thereby influencing their performance in various applications. For instance, Ali *et al.*^49^ showed that by varying annealing temperature, Cu_2_MoS_4_ nanosheets could be transformed into MoS_2_ and Cu_2_S nanoheterostructures, resulting in enhanced specific charge capacity and improved cycling stability as anode materials for Li-ion batteries. Similarly, Abbas *et al.*^50^ reported that the annealing temperature directly impacts the morphology and microstructure of Ti–Al–C thin films on stainless steel substrates. These findings underline the broader importance of fine-tuning annealing temperatures to conductivity in various systems, including our work with PDCs.

**Fig. 1:**
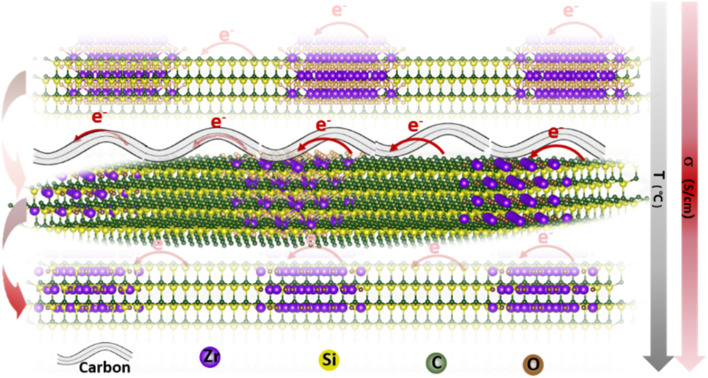
Schematic illustration of the electron transport route within the SiC-based ceramics under different formations.

Herein, a novel method for synthesizing polycarbosilane (PVZCS) modified with unsaturated vinyl group and zirconium is introduced to systematically enhance the electrical conductivity of SiC-based ceramics. The polymer-to-ceramic transformation and the nano-microstructural evolution are investigated, focusing on how these changes affect electron migration and conductivity. The presence of free carbon, observed *via* electron microscopy, and its impact on conductivity are thoroughly examined. As shown in Fig. 1, as the temperature increases, the conductivity of SiC-based ceramics reaches its peak at 1400 °C. This peak is attributed to the evolution of the free carbon distribution and the enhanced electron migration behavior. The SiC-based ceramic with the optimized condition behaves quite well in LIBs anode application, proved by the LIBs cycling operation experiments. Additionally, density functional theory (DFT) is employed to calculate the density of states (DOS) with a gradient carbon percentage, giving the theoretical support of the conductivity enhancement observed from our experimental results. This study not only elucidates the vital importance of free carbon content and distribution on the electrical conductivity of PDCs, but also opens new avenues for the application of Si-based ceramics in a multitude of high-tech domains.

## 2. Results and discussion

**Fig. 2:**
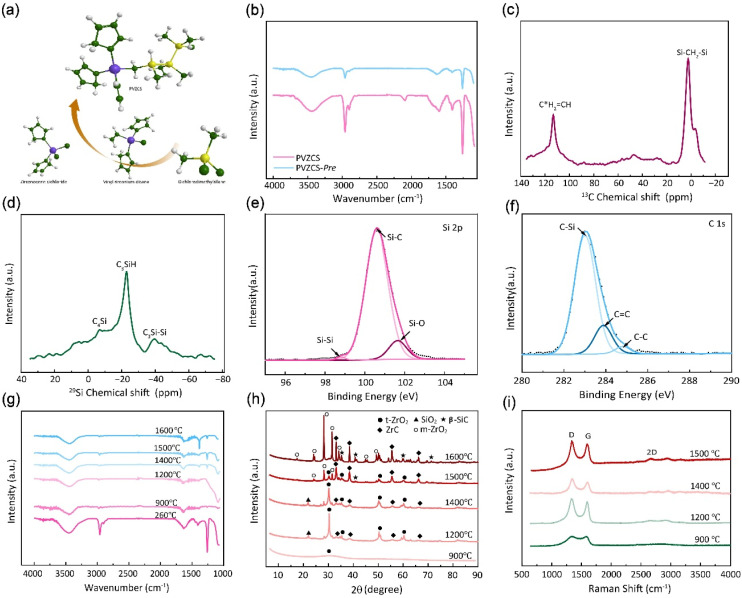
(a) Schematic illustration of the preparation process of PVZCS. (b) FTIR infrared spectroscopic analysis of PVZCS and PVZCS-Pre. (c) Nuclear magnetic resonance spectroscopy spectrum of ^13^C-NMR and (d) spectrum of ^29^Si-NMR for the molecular. (e) Si 2p XPS spectrum and (f) C 1s spectra of indicated PVZCS. (g–i) FTIR spectra, XRD pattern and Raman plot of the specimen subjected to pyrolysis and annealing at temperatures ranging from 900 °C to 1600 °C.

In Fig. 2a, a comprehensive elucidation of the molecular structure, chemical characterization properties pertaining to PVZCS is provided, with the FTIR spectroscopy illustrated in Fig. 2b. Resonance identified at 3099 cm^−1^ elucidates the involvement of raw material Cl_2_Cp_2_Zr in the synthesis. A distinct absorption peak at 3067 cm^−1^ is emblematic of the presence of active vinyl functional moieties. Concurrently, a series of peaks serve as evidence of the complex interplay among structural units, as illustrated below: 2963 cm^−1^ (*ν*, C–H stretching in –Si–CH_3_), 1408, 1262 cm^−1^ (*δ*, Si–C deformation in –Si–CH_3_), 2901 cm^−1^ (*ν*, C–H stretching in –Si–CH_2_–Si–), 1049 cm^−1^(*ν*, Si–C stretching in –Si–CH_2_–Si–), 2093 cm^−1^ (*ν*, Si–H stretching)and 1598 cm^−1^ (*ν*, C=C stretching). To encapsulate, the amassed infrared spectroscopic data proffers comprehensive information on PVZCS, emphasizing its intricate structures.

The synthesized PVZCS was annealed at 260 °C/2 h to induce thermal cross-linking, thereby elevating the molecular weight (denoted as PVZCS-Pre). As discernible from the figure, there is a marked diminution in the absorption peak corresponding to the –Si–H bond relative to the untreated specimen. The alteration in the –Si–H bond peak serves as an indicator of the degree of thermal cross-linking. In proximity to 400 °C, –Si–CH_3_ undergoes a demethylation reaction with –Si–H, resulting in the evolution of methane gas and the formation of –Si–Si– bonds. Given this knowledge, it is postulated that the characteristic peak of –Si–CH_3_ remains largely invariant during the thermal cross-linking process at 260 °C. Conclusively, the thermal cross-linking extent is quantitatively represented by the ratio of the characteristic peaks of –Si–CH_3_ to –Si–H, denoted as *P*_Si–H,_ which is mathematically expressed in eqn (1). A *P*_Si–H_ value of 89% suggests a substantial degree of thermal cross-linking post the 260 °C/2 h process. Predominant reactions during this phase encompass dehydrogenation coupling, as described in reaction (2), and the hydrosilylation reaction, as elucidated in reaction (3).1
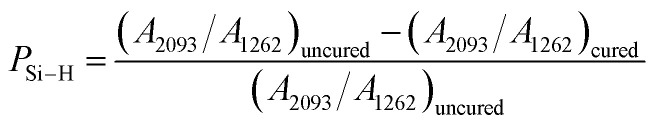

2 –Si–H + –Si–H → –Si–Si– + H_2_

3 –CH=CH_2_ + –Si–H → –CH_2_–CH_2_–Si–

4 –Si–CH_3_ + –Si–H → –Si–Si– + CH_4_

The molecular architecture of the sample is further elucidated using NMR spectroscopy. Fig. 2c presents the solid-state ^13^C-NMR spectrum, where a dominant signal peak attributed to –Si–CH_2_–Si– appears at *δ* = 0 ppm. Signals detected in the ranges of 20–30 ppm and 40–50 ppm indicate the presence of –Si–CH_3_ groups. Notably, resonances at 113 ppm and 133 ppm are characteristic of –CH=C*H_2_ and –C*H=CH_2_ groups. In the solid-state ^1^H-NMR spectrum (Fig. S1†), the peak centred at 0 ppm corresponds to –Si–CH_2_–Si–, while the broad peak ranging from 5–7 ppm is assigned to –CH=CH_2_ group. Fig. 2d illustrates the solid-state ^29^Si-NMR spectrum, where resonances within the ranges of −10–0 ppm, −25 to −15 ppm, and −45 to −35 ppm are ascribed to –C_4_Si, –C_3_SiH, and –C_3_Si–Si– respectively. This data confirms the presence of both –Si–CH_3_ and –Si–CH_2_–Si– structural motifs in the backbone, along with –CH=CH_2_ groups.

The elemental composition and the overall electronic structure of PVZCS are identified through XPS. As illustrated in Fig. 2e, f, S2 and S3†, predominantly features the elements C, O, Si, and Zr. The Si 2p spectrum is characterized by three peaks: 98.8 eV, corresponding to the –Si–Si– bond; 100.6 eV, associated with the –Si–C– bond; and 101.7 eV, indicative of the –Si–O– bond. The presence of the Si–Si bond at 98.8 eV suggests a dehydrogenation coupling reaction occurring in the sample. The C 1s spectrum unveils three characteristic peaks with binding energies at 283.0 eV, 283.9 eV, and 284.8 eV, which correspond to the –C–Si, –C=C–, and –C–C– bonds, respectively. The –C=C– bond arises from both the dicyclopentadienyl ring linked to the zirconium atom and the –CH=CH_2_ group directly bonded to Zr, a result of the Grignard coupling reaction during synthesis. For the O 1s spectrum, three deconvoluted peaks are observed at 529.0 eV, 530.6 eV, and 531.7 eV, which are attributed to Zr=O, Si=O, and C=O, respectively.

Fig. S4† depicts the TGA-DSC profile of PVZCS ceramicization, which can be divided into four distinct stages. The initial stage (room temperature to 250 °C) results in a 7 wt% weight loss due to the evaporation of water and minor organic compounds. In the intermediate stage (250 °C to 800 °C), a 25 wt% weight reduction is accompanied by an exothermic peak at 430 °C, reflecting dehydrogenation coupling and hydrosilylation reactions, followed by an endothermic demethanation reaction, as described in reaction (4). Minimal weight, loss (1 wt%) characterizes the third stage (800 °C to 1100 °C), concluding PVZCS pyrolysis and zirconium dioxide (ZrO_2_) crystallization. Beyond 1100 °C an additional 6 wt% mass reduction is observed, with an endothermic peak at 1250 °C, attributed to CO and CO_2_ evolution and ZrO_2_ to zirconium carbide (ZrC) transition. This concise analysis elucidates the thermal behaviour processes.

The FTIR spectra and XRD pattern of PVZCS are examined within the pyrolysis range of 900 °C to 1600 °C, in alignment with prior TGA-DSC findings indicating significant crystallization above 800 °C (Fig. 2g and h). Fig. 2g clearly shows that certain characteristic peaks, such as 2963 cm^−1^, 1408 cm^−1^, and 1262 cm^−1^(associated with Si–CH_3_), disappear at 900 °C. The XRD pattern further reveals the crystal evolution of PVZCS as the temperature increases from 900 °C to 1600 °C. At 900 °C, a broad peak at 2*θ* = 30° signifies the presence of amorphous ZrO_2_, indicating Zr interacts with O. At 1200 °C, well-defined diffraction peaks corresponding to the tetragonal crystalline phase (*t*-ZrO_2_) (JCPDS #81-1544; 30.1° and 50.2°) emerge, along with the characteristic diffraction peaks of ZrC (JCPDS #65-0332; 33.0°, 38.3°, 55.3°, 65.9°, and 69.2°) and SiO_2_ (JCPDS #29-0085; 22.0°). Intriguingly, as temperature climbs to 1500 °C, diffraction peaks at 28.2° ((−111) facets) and 31.5° ((111) facets) appeared, indicating a phase transformation into monoclinic crystalline phase (*m*-ZrO_2_) (JCPDS #65-1025; 17.5°, 24.2°, 28.2°, 31.4° and 50.1°).^51^ As temperature further increases, *t*-ZrO_2_ peaks diminish, completely disappearing at 1600 °C, while ZrC peaks and *m*-ZrO_2_ peaks intensify. Typically, zirconium in its pristine state exhibits *m*-ZrO_2_ phase at room temperature. When subjected to the high temperature of 1173 °C, it undergoes a phase transition into a *t*-ZrO_2_ phase. Further elevation of the temperature to around 2370 °C prompts a transformation into a cubic crystalline phase. Nevertheless, numerous investigations have indicated that under high-temperature conditions, specifically as suggested by Ding,^52^ the *t*-ZrO_2_ tends to revert to a monoclinic configuration. It is reasonable to postulate that within the lower thermal range (900–1200 °C), zirconium predominantly forms ZrO_2_ through oxygen interaction; within 1200–1400 °C, the amorphous ZrO_2_ transformed to crystalline *t*-ZrO_2_. After 1500 °C, a portion of *t*-ZrO_2_ phase enters a chemical reaction with carbon, forming ZrC aligning with TGA curve trends post-1200 °C, while another portion reverts into *m*-ZrO_2_. Around 1200 °C, a SiO_2_ peak emerges at 2*θ* = 22°, receding at 1500 °C, while SiC (JCPDS #29-1129; 35.6°, 41.5°, 60.0°, 71.8° and 74.5°) peaks begin to appear in the resultant product.

The structural evolution of the segmented carbon phase, commonly referred to as “free carbon”, is preliminary assessed using Raman spectroscopy (Fig. 2i). There is a pronounced D peak at around 1350 cm^−1^, which is linked to the breathing modes of sp^2^ carbon atoms in rings, and a G peak at roughly 1585 cm^−1^, tied to in-plane bond stretching of sp^2^ carbon.^53^ The *I*_D_ intensity signifies a disordered carbon network, while the *I*_G_ intensity portrays a graphitic network. A higher *I*_D_/*I*_G_ indicates greater free carbon disorder. The 2D band reflects changes in the electronic structure. Data from Table 1 demonstrates that with a treatment rise from 900 °C to 1200 °C, the D-to-G peak intensity ratio (*I*_D_/*I*_G_) first drops from 1.29 to 1.10, before climbing to 1.27. The fluctuation in the *I*_D_/*I*_G_ ratio suggests that the graphitization degree of the structure reaches a pivotal point during this trend. The ratio also offers insights into the dimensions of free carbon lateral size on the sixfold ring plane, symbolized as La.^54^ For highly disordered graphite, the La can be derived from the Ferrari–Robertson equation.5
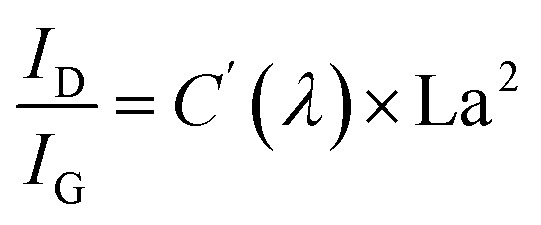
where *C*′(*λ*) = *C*_0_ + *λC*_1_ (*C*_0_ = −12.6 nm and *C*_1_ = 0.033) and *λ* represents the excitation source wavelength. Observations show that the size diminishes when the treatment is below 1200 °C but rises at 1400 °C. This behaviour is succinctly explained by the Ferrari Model. Initially, the decrease in lateral size at lower temperatures results from the sp^3^–sp^2^ transition and the reformation of distorted aromatic rings into six-membered ones. Subsequently, the size increase arises from the in-plane expansion of nano-graphite. These findings, combined with the narrowing of both the D and G peaks FWHM, indicate a transition of the free carbon phase from amorphous carbon to nanocrystalline graphite as thermal treatment temperature escalates.

**Table 1:** Raman spectroscopy data of the specimen annealing at different temperatures

Samples	*I*_D_/*I*_G_	D peak		G peak		La (nm)
		Location (cm^−1^)	FWHM (cm^−1^)	Location (cm^−1^)	FWHM (cm^−1^)	
PVZSC-900	1.29	1365	298.9	1581	113.0	0.260
PVZSC-1200	1.10	1339	167.4	1587	100.9	0.222
PVZSC-1400	1.27	1353	145.4	1591	117.3	0.256
PVZSC-1500	1.12	1350	162.6	1589	110.4	0.226

This transformation entails the evolution and fate of amorphous free carbon to nano-crystalline graphite, and it gradually occurs with increasing temperature. For thermal treatment lower than 900 °C, excess carbon has only begun to segregate and is amorphous, hydrogenated, and still dispersed in the ceramic matrix.^3,55^ Increasing thermal treatment to 1000 °C results in a “free carbon” phase described as 2–3 layers thick turbostratic carbon saturated with hydrogen at the periphery. Pyrolysis temperatures higher than 1000–1200 °C eliminate hydrogen from peripheral carbons and yield extended carbon layers. Above pyrolysis temperatures of 1200 °C, the excess carbon phase forms graphite nano-crystallites or an entangled network of single or multi-layered graphene. As can be seen from the result, the 2D peak appears when materials pyrolysis above 1400 °C, indicating that free carbon stacking occurs inside the material.

**Fig. 3:**
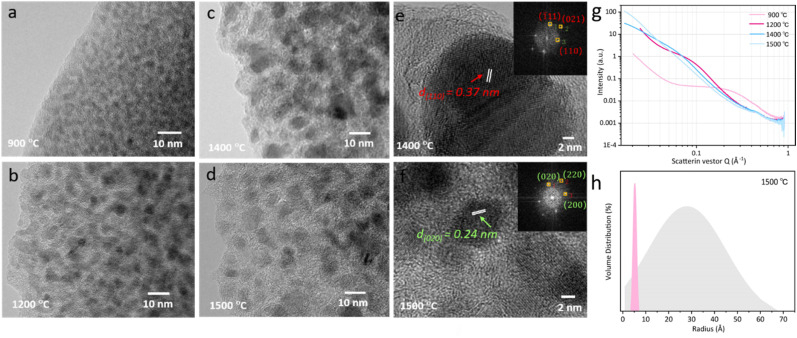
(a–d) TEM images of PVZCS annealed at varying temperatures. (e and f) HRTEM of the sample annealed at 1400 °C and 1500 °C, with the SAED in the insets, respectively. (g) SAXS spectroscopy pattern of the PVZCS. (h) Size distribution of the particle at 1500 °C.

The microstructures of the sample are characterized using a TEM. Fig. 3a–d illustrate the nanostructure of the nanocomposite phase. At 900 °C, the structure is amorphous. However, upon reaching 1200 °C, the crystallization starts to emerge. The size of these crystal phase particles expands as reaches 1400 °C. Beyond 1500 °C, heterogeneous smaller particles begin to appear on the horizon. The results correspond with the XRD results depicted in Fig. 2h well, indicating the uniformly distributed phase at the initial temperature of 900 °C is ZrO_2_ amorphous phase. The subsequent rise in temperature promotes the phase transition from the amorphous state to the crystalline *t*-ZrO_2_ phase. Once this transformation is completed at 1400 °C, the crystals enter a growth phase. Concurrently, another crystal phase, ZrC, begins to manifest. As a result, a significant amount of the ZrO_2_ crystal phase with larger size can be observed, accompanied by a small fraction of the ZrC crystal phase with tinier size. High-resolution TEM (HRTEM) images of PVZCS annealed at 1400 °C further highlight the crystalline structure of *t*-ZrO_2_ in the (110) facets, exhibiting a typical spacing of *d* = 0.37 nm (see Fig. 3e). For samples annealed at 1500 °C, PVZCS displays a lattice spacing of *d* = 0.24 nm, corresponding to the (020) direction of ZrC (refer to Fig. 3f). This observation aligns perfectly with the previously mentioned XRD test results.

Alterations in the crystal structure encompass not only variations in the types of phases but also modifications in crystal dimensions. Small angle X-ray scattering (Fig. 3g) is utilized to scrutinize the internal architecture in depth. Our SAXS analysis supposed that the crystal particles within the sample are an ellipsoidal shape. The particle size is deduced using the equation *q* = 4π sin *θ*/*λ*, where *λ* is the X-ray wavelength and *θ* is one-half the scattering angle. Across the temperature range of 900 °C to 1500 °C, the respective crystal particle dimensions are 0.7 nm, 1.44 nm, 1.47 nm, and 3.92 nm. Evidently, upon reaching 1500 °C, there is a pronounced shift in the internal particle dimensions. A statistical examination of the internal particle distribution at this temperature was undertaken. As depicted in Fig. 3h, the sample internal particle size distribution spans less than 6 nm, with a mean size of approximately 3 nm. Concurrently, there is a discernible distribution of minuscule particles, characterized by a narrow peak width, measuring around 1 nm.

Correlating this with the above discussions, below 1400 °C, the consistent enlargement of crystal dimensions is attributable to the growth of the *t*-ZrO_2_ crystal phase. However, upon achieving 1500 °C, the system witnesses a comprehensive transformation from the *t*-ZrO_2_ to *m*-ZrO_2_ and ZrC, leading to a substantial uptick in particle dimensions. Simultaneously, the emergence of smaller sized SiC crystals is formed and observed at 1600 °C (Fig. S5†).

**Fig. 4:**
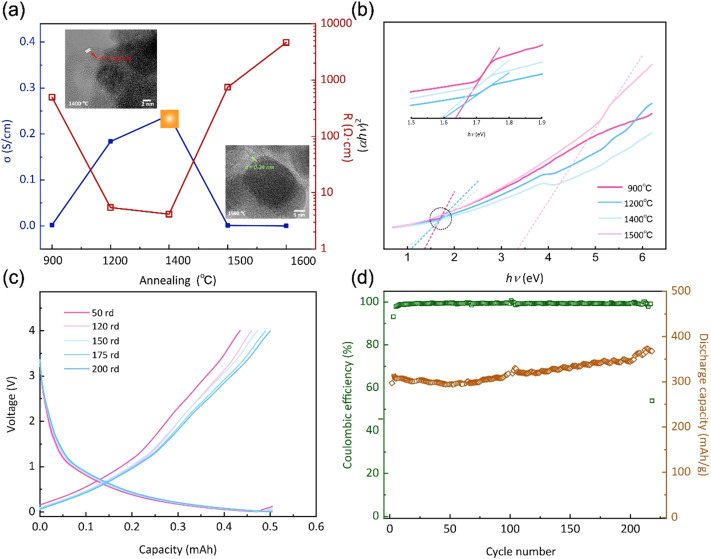
(a) Conductivity and resistance and (b) bandwidth spacing of the annealed specimen. (c) Charge–discharge and (d) cycle stability of Li-ion battery performance with assembled this material as anode.

Furthermore, when subjected to treatment at 1400 °C, the expansion and reorganization of free carbon regions persist, ultimately leading to the formation of an intricate network or nanocluster composed of graphitic nano-crystallites. Under these conditions, the presence of a ribbon-like carbon structure with well-defined lattice fringes and inter-planar spacing becomes apparent. As illustrated in the HRTEM image presented in Fig. 4a, the carbon ribbon exhibits a distinctive spacing of 0.34 nm, clearly visible and indicative of its crystalline nature. These ribbons manifest in a relaxed configuration and are uniformly coiled along the peripheries. However, subjected to treatment at 1500 °C, these ribbons vanish from the edges, only to re-emerge along the crystal boundaries. Intriguingly, at this temperature, these ribbons are tautly wrapped around the boundaries, enveloping the internal crystal. The results indicate that the progressive increase of the temperature shifts the locations of these free carbon ribbons, as well as affects the structural equilibrium position, leading to changes in electron migration behaviour.

The spatial distribution and morphology of free carbon within PVZCS are not incidental characteristics but rather fundamental determinants of the material electronic behaviour. This interplay becomes particularly salient when examining the intricate parabolic resistance pattern illustrated in Fig. 4a. A remarkable peak in electron transport efficiency, reaching 0.28 S cm^−1^, is attained at 1400 °C. There are two primary factors contributing to this transformation. Firstly, this optimal conductivity is largely influenced by the transformation of the Zr phase: the material progresses from an amorphous ZrO_2_ structure, transitions into its *t*-ZrO_2_ phase(<1400 °C), and subsequently transforms into ZrC and *m*-ZrO_2_ phases (>1500 °C). The presence of *t*-ZrO_2_ significantly enhances conductivity. However, with the phase transition to *m*-ZrO_2_, conductivity experiences a substantial decline due to the inherently lower conductivity. Despite the concurrent high conductivity of ZrC, the prevailing influence is exerted by the *m*-ZrO_2_ layer covering the surface of ZrC. Second, the structural changes in Zr directly impact the spatial distribution and morphological alterations of free carbon, thereby reconfiguring the conductive network within the material. The extensive dataset, spanning a range from 2.15 × 10^−7^ S cm^−1^ to 0.28 S cm^−1^ in a more conservative estimation, underscores the profound influence of precise annealing on conductance.

The forbidden bandwidths of the material were examined to further validate our hypothesis. The rate of diffuse reflection coefficient varies for the four specimens in the low energy region, allowing us to deduce that each specimen possesses distinct forbidden bandwidths (Fig. S6†). The Tauc plot method aids in pinpointing the absorption edge, subsequently facilitating the determination of the semiconductor forbidden bandwidth, expressed as:

6 (*αhν*)^2^ = *A*(*hν* − *E*_g_)

Here, *α* represents the absorbance coefficient, *h* is Planck's constant, *ν* is the frequency of the light wave, *A* is a constant, and *E*_g_ is the forbidden bandwidth. The relationship between (*αhν*)^2^ and *hν* is illustrated in Fig. 4b. The derived *E*_g_ for the four specimens are as follows: 1.58 eV, 1.48 eV, 1.47 eV, and 3.61 eV. As the treatment climbs to 1400 °C, there is a consistent decrement in the *E*_g_, enhancing the electrical conductivity. However, further escalation in the thermal treatment leads to a stark augmentation in the *E*_g_. In essence, the intricate interplay of thermal treatment, phase transitions, and resultant free carbon dynamics underscores the significant potential and challenges in harnessing and optimizing PVZCS conductivity properties for advanced applications.

Based on the preceding investigations, the PVZCS treated at 1400 °C exhibited comparable high conductivity. Subsequently, an evaluation of its electrochemical performance as a Li-ion battery electrode material was conducted. The sustained cycling performance is depicted in Fig. 4c and d, highlighting a specific capacity of 383.5 mA h g^−1^ and a cyclic CE of 98%. Remarkably, obvious evidence of capacity increase is observed throughout the cyclic tests, which is often observed in the early stage of Li-ion battery cycling. This special phenomenon is mainly attributed to material activation and the increase of the *d*-spacing of graphite during cycling. Since the Li-ion battery performance assessments conducted in this study utilized materials subjected to pure thermal treatment, without any further optimization or modifications intended for enhancing battery performance. Consequently, direct comparisons of parameters, such as specific capacity, with high-performance data reported in existing literature may not be entirely justified. Nevertheless, our material demonstrates exceptional CE and robust cyclic performance. This resilience underscores a fundamental characteristic of silicon-based electrode materials: their inherent high stability.

**Fig. 5:**
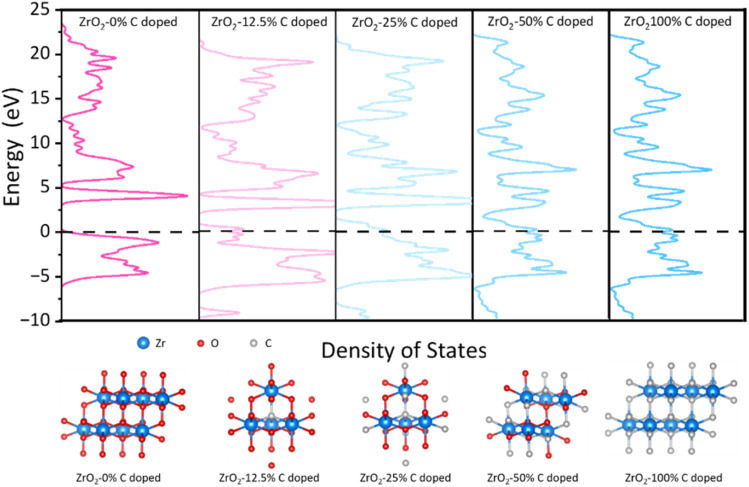
Density of states and the relaxed structures of all ratios of C doped species by DFT calculations. The values (eV) are the relative energies (Δ*E*) to Fermi level.

Finally, based on DFT calculations, first principle calculations were undertaken to discern the influence of carbonization degrees on the electrical conductivity of the ZrO_2_ structure and rigorously explore the implications of the Zr-phase transformation, specifically the alterations in the spatial distribution and morphology of free carbon ribbons on electron transport properties. As presented in Fig. 5, the undoped ZrO_2_ cell exhibits pronounced semiconducting characteristics with an energy gap of 3.2 eV. However, with 12.5% carbon doping in ZrO_2_, this energy gap diminishes to approximately 1 eV. Further doping reduces the energy gap of the structure to nearly 0 eV, as observed in the ZrO_2_ with 25% carbon doping. This structure evolves into a commendable conductor as the concentration of doped free carbon in the lattice escalates. Our result shows that increasing temperatures lead to the lattice transition from ZrO_2_ to ZrC, potentially merging the energy bands between the free carbon ribbon and the native cell. This fusion might result in the narrowing of the energy gap, prompting the lattice to assume conductive behaviour. These findings hint that annealing at an optimal temperature, such as PVZCS at 1400 °C, could optimize the electron transport property of the system.

## 3. Experimental sections

### 3.1 Materials

Zirconocene dichloride (Cp_2_ZrCl_2_, 98%), *n*-pentane (C_5_H_12_, 99%), dichlorodimethylsilane (Me_2_SiCl_2_, >98.5%), and sodium (Na, 98%) were purchased from Shanghai Aladdin Biochemical Technology Co., Ltd. Xylene (C_8_H_10_, 99%), petroleum ether and vinyl magnesium chloride (ViMgCl) solution of 1.6 mol L^−1^ in tetrahydrofuran was obtained from Shanghai Macklin Biochemical Co., Ltd. Toluene (C_7_H_8_, 99.8%) was provided by Xilong Scientific Co., Ltd.

### 3.2 Synthesis of polyvinylzirconocarbosilane (PVZCS)

All operations were executed in glassware that had been pre-dried in an oven, with all manipulations taking place under a blanket of inert gas. To initiate the reaction, a 500 mL three-necked flask was loaded with 250 mL of xylene, Cp_2_ZrCl_2_, and ViMgCl under a nitrogen atmosphere, maintaining a molar ratio of 1 : 0.5. This specific ratio was established as optimal following a series of preliminary trials according to yield of eventual products, reaching 45.55% when the ratio of Me_2_SiCl_2_ and Vi-zirconocen maintained 5 : 1. The reaction admixture was stirred at 0 °C for a duration of 4 hours and then at ambient temperature overnight. Subsequently, the reaction was terminated *via* vacuum filtration, and the resulting filtrate underwent extraction with pentane. Following the removal of the solvent through filtration, the isolated products were subjected to a drying process under vacuum at 60 °C. After carrying out these operations, experimental intermediate products, Vi-zirconocene were obtained.

During the second stage of synthesis, a 500 mL four-necked flask, equipped with a reflux condenser, dropping funnel, and nitrogen inlet/outlet was prepared. In this setup, 250 mL of toluene and a slight excess of sodium were initially introduced. Under continuous heating and stirring, the sodium chunks melted and formed droplets dispersed in toluene at 90 °C under a nitrogen atmosphere. Subsequently, Me_2_SiCl_2_ and Vi-zirconocene, with a fixed molar ratio of 3 : 1, were added to the solution and allowed to react for 10 hours. Molar ratios of 2 : 1, 5 : 1, and 8 : 1 were tested but found to be inferior to 3 : 1 ratio, based on the yield and molecular weight of the final products. The reaction was completed by the addition of ammonia, which also helped stabilize the resulting product. The solvent was removed *via* suction filtration, and the precipitate obtained was the target product, polyvinylzirconocarbosilane (PVZCS).

### 3.3 Thermal treatment and pyrolysis of PVZCS

The PVZCS samples underwent a post-synthesis thermal treatment to enhance their properties. The procedure involved heating the samples to 260 °C for 2 hours under an argon gas atmosphere, with a controlled temperature increase of 5 °C min^−1^.

The samples were progressively heated to predetermined temperatures of 900 °C, 1200 °C, 1400 °C, 1500 °C, and 1600 °C for 2 hours. The materials obtained at the conclusion of the pyrolysis process were named in correspondence with the final temperature they were exposed to, resulting in PVZCS-900, PVZCS-1200, PVZCS-1400, PVZCS-1500, and PVZCS-1600. These specimens were then meticulously analyzed to determine their thermal stability and the efficiency of their conversion to ceramic.

### 3.4 Characterization

Fourier-transform infrared (FT-IR) spectra were acquired using a Bruker TENSOR27 spectrometer, employing KBr discs to analyze the samples. Proton (^1^H), carbon (^13^C), and silicon (^29^Si) nuclear magnetic resonance (NMR) spectra were recorded on a Bruker AVANCE III 600 spectrometer, with tetramethylsilane (TMS) serving as the external standard for chemical shift referencing. Thermalgravimetric analysis coupled with differential scanning calorimetry (TG-DSC) was performed on a PerkinElmer STA 6000 instrument. The samples were heated from 30 °C to 1400 °C at a rate of 10 °C min^−1^ under an argon atmosphere, providing insights into their thermal stability and decomposition behaviour. The crystalline structure and phase purity of the samples were investigated using X-ray diffraction (XRD) on a Smartlab-9 system from Rigaku. Raman spectra were obtained using an inVia confocal Raman microscope spectrometer from Renishaw, with a laser excitation wavelength of 532 nm. The surface chemical states of the samples were probed using X-ray photoelectron spectroscopy (XPS) on an AXIS SUPRA^+^ instrument from Shimadzu. The microstructure and morphology of the synthesized samples were examined using Hitachi Regulus8100 scanning electron microscopes (SEM) and a JEM-2100F transmission electron microscope (TEM) from JEOL. This high-resolution imaging allowed for the detailed observation of the samples at the nanometer scale. Small angle X-ray scattering experiments were conducted using the Xeuss 2.0 system by Xenocs, with a copper target X-ray source operating at 30 W and a wavelength of 1.54189 Å. The Pilatus 3R 300K detector, featuring a pixel size of 172 μm, was used to capture the scattered X-rays, ensuring high-resolution data for structural analysis. The optical properties of the materials were characterized by UV-vis diffuse reflectance spectroscopy (DRS) using a UV-3600i Plus spectrometer. Absorption spectra were recorded over the wavelength range of 200–800 nm to determine the band gap energies. The correlation between the diffuse reflection coefficients of specimens subjected to different annealing temperatures and the photo wavelength. The electrical conductivity of the materials was measured using the four-point probe method using a FT-201A instrument. This technique was chosen to minimize contact resistance and to provide an accurate assessment of the intrinsic material properties.

The synthesized materials were evaluated as potential anode materials for lithium-ion batteries. The preparation details are as follows: A mixture containing 80 wt% of the active materials obtained from experimental section, 10 wt% of conductive carbon black, and 10 wt% of polyvinylidene fluoride (PVDF) was prepared and ground together, using 1-methyl-2-pyrrolidinone (NMP) as a solvent for homogeneous mixing. The obtained slurry was coated onto a copper foil and dried under vacuum conditions for 10 hours to remove excess NMP. After drying, the material was sliced into 12 mm diameter discs serving as anode materials, with lithium metal used as the counter electrode. Galvanostatic charge–discharge testing was performed using CT-3002A measurement system within a voltage range of 0.01 V to 4 V.

Density Functional Theory (DFT) computations for the investigation of doped species and their electronic structures were executed utilizing the CASTEP computational code. The exchange–correlation interactions intrinsic to the systems under study were modelled using the Generalized Gradient Approximation (GGA) with the Perdew–Burke–Ernzerhof (PBE) functional, a widely acknowledged approach for the treatment of electronic density gradients. A cutoff energy of 400 eV was selected for the plane-wave basis sets, a parameter critical for the precision of total energy calculations. The geometry optimizations of the structures were carried out employing the Broyden–Fletcher–Goldfarb–Shanno (BFGS) algorithm, a robust method for finding the nearest local minimum on the potential energy surface. For the determination of total energy convergence, a Monkhorst–Pack grid of *k*-points was established at a density of 8 × 8 × 8. This ensured the total energy of the system was converged to a threshold below 1.0 × 10–6 eV per atom, signifying a high level of precision in the electronic structure calculations. Additionally, the Hellmann–Feynman forces acting upon the atoms converged to a criterion of less than 0.03 eV Å^−1^, thereby confirming the attainment of a stable optimized geometry with minimal residual forces.

## 4. Conclusions

In conclusion, our work systematically showed the remarkable potential of SiC-based ceramics synthesized from a vinyl/zirconium-modified polycarbosilane precursor. Through meticulous control of nanoscale properties and controlled thermal treatment, a material with exceptional pinpoint electrical conductivity and minimal forbidden bandwidth has been unveiled. The comprehensive analysis elucidates the intricate structural evolution as amorphous ZrO_2_ transforms into distinct phases, including *m*-ZrO_2_, *t*-ZrO_2_, and ZrC, thereby shedding light on the consequential fate of “free carbon”. The comprehensive structural analysis, including the lattice transition and the influence on “free carbon” provides valuable insights into the material behaviour. Furthermore, our findings, supported by the theory calculations with DFT analysis, suggest a novel mechanism wherein the energy bands of carbon merge with those of the native cell, resulting in a narrowed energy bandgap and enhanced conductivity. The constructed lithium-ion battery demonstrates outstanding electrochemical performance, affirming its potential as a stable and efficient anode material. Our work creatively synthesized PVZCS with the unsaturated vinyl group and zirconium, which not only highlights the importance of structural modification for Si-based advanced ceramics but also paves the way for their practical application in cutting-edge systems.

## Data availability

The data supporting this article have been included as part of the ESI.†

## Author contributions

All authors discussed the results and contributed to the final manuscript. JJ. Jiang carried out the experiment. SW. Ying developed the theory and performed the computations. CX. Wu and C. Yang devised the project, the main conceptual ideas and proof outline.

## Conflicts of interest

There are no conflicts to declare.

## Supplementary Material

RA-014-D4RA06633B-s001
