# Supplementary material for: Enhancing electrical conductivity in zirconium-doped SiC ceramics through synergistic effects of crystal structure and free carbon control
Source: RSC Adv. 2024 Oct 28;14(46):34328–37. doi: 10.1039/d4ra06633b (PMC11514724; doi:10.1039/d4ra06633b)
Supplement: RA-014-D4RA06633B-s001 [file RA-014-D4RA06633B-s001.pdf]

Supplementary Information (SI) for RSC Advances.
This journal is © The Royal Society of Chemistry 2024

Supporting Information

Enhancing Electrical Conductivity in Zirconium-Doped SiC Ceramics through Synergistic Effects of Crystal Structure and Free Carbon Control

Jiaojiao Jiang [a,b], *Siwei Ying* [d], *Chunxiao Wu* [a,b,c*], *Chao Yang* [a,b*]

a. State Key Laboratory of Biochemical Engineering

Key Laboratory of Biopharmaceutical Preparation and Delivery

Key Laboratory of Green Process and Engineering

Institute of Process Engineering, Chinese Academy of Sciences

Beijing, 100190, China

E-mail: cxwu@ipe.ac.cn; chaoyang@ipe.ac.cn

b. School of Chemical Engineering

University of Chinese Academy of Sciences

Beijing, 100190, China

c. Hubei Three Gorges Laboratory.

Yichang, 443100, China

d. State Key Laboratory of Physical Chemistry of Solid Surfaces

Collaborative Innovation Center of Chemistry for Energy Materials (iChEM)

College of Chemistry and Chemical Engineering

Xiamen University

Xiamen, 361000, China

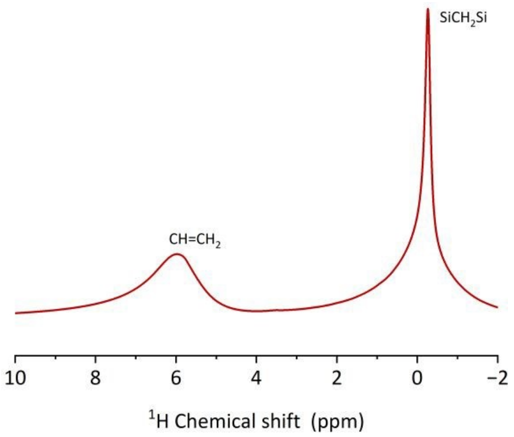

Fig. S1 Nuclear magnetic resonance spectroscopy spectrum of ^{1}H-NMR for the molecular.

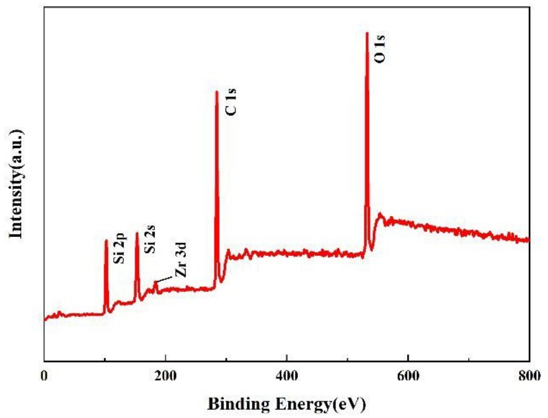

Fig. S2 The XPS full elements exhits in the PVZCS, with atomic proportions of 55.6 wt%, 23.82 wt%, 20.04 wt%, and 0.54 wt%, respectively.

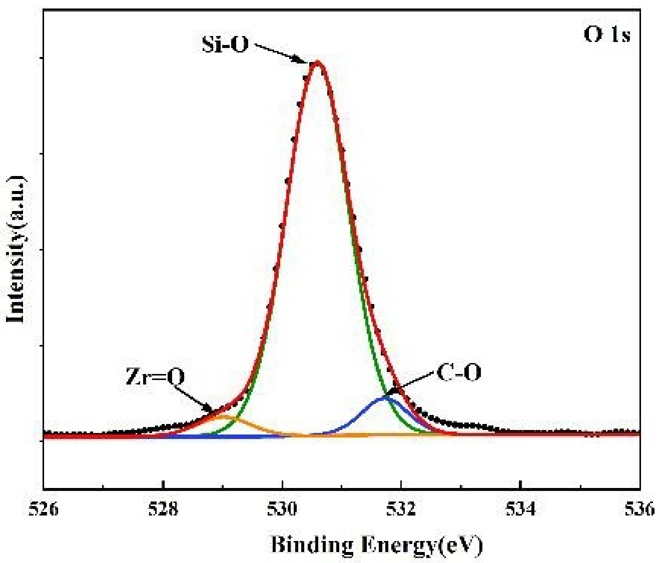

Fig. S3 O 1s XPS spectrum spectra of indicated PVZCS.

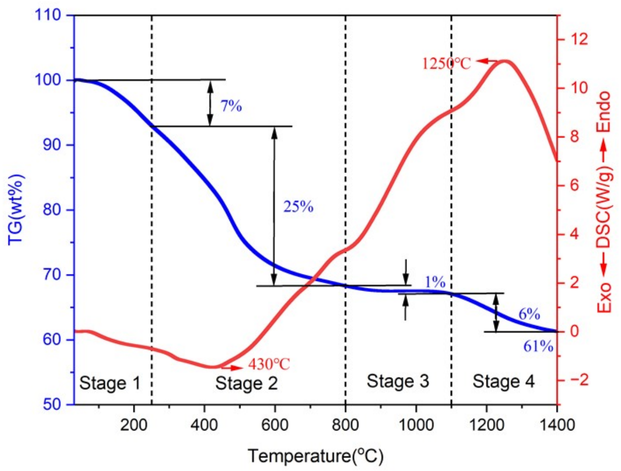

Fig. S4 TGA-DSC profile of PVZCS.

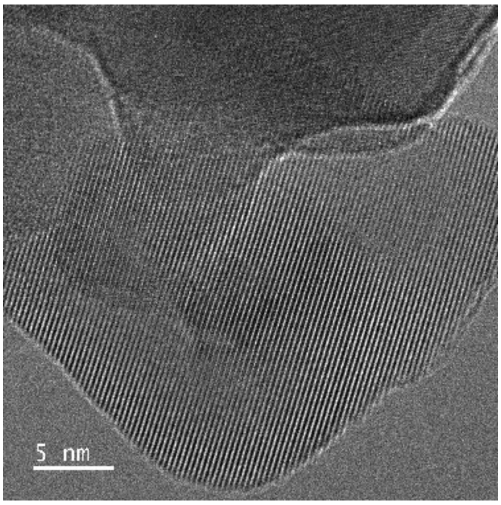

Fig. S5 TEM image of PVZCS pyrolysis at 1600°C

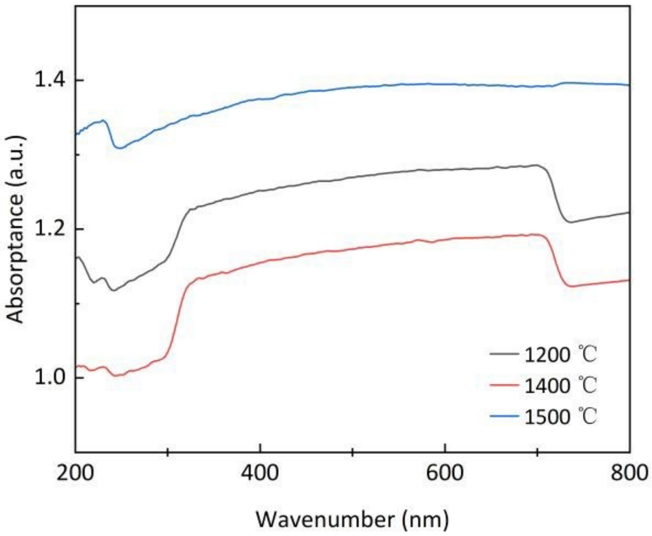

Fig. S6 The UV spectrum of the samples.
